# Backslide or forward progress? Virtual care at U.S. healthcare systems beyond the COVID-19 pandemic

**DOI:** 10.1038/s41746-020-00379-z

**Published:** 2021-01-08

**Authors:** Spencer D. Dorn

**Affiliations:** Department of Medicine, University of North Carolina School of Medicine, Chapel Hill, NC USA

**Keywords:** Health care, Health services

## Abstract

The COVID-19 pandemic forced most U.S. healthcare systems to quickly pivot to virtual care. However, since peaking in late April, care has largely shifted back to in-person. Health systems are now challenged to further develop and integrate useful, usable, and sustainable virtual care tools into their broader care model in ways that benefit their organizations and the communities they serve.

At the start of 2020, very few U.S. healthcare systems had embraced virtual care at scale^1^. Incentives to use these tools were generally not strong enough to overcome considerable barriers to change. By March, in the face of the COVID-19 pandemic, most systems quickly pivoted to virtual care to preserve care access, protect their patients, sustain their workforces, and maintain revenue. Within weeks, most systems were providing more virtual care—including video and phone visits, eVisits, eConsults, and messaging with clinicians and chatbots—in a single day than they had the entire prior year.

However, since peaking in late April, care has largely shifted back from virtual to in-person^2,3^, sometimes because in-person care is more clinically appropriate, but often simply because it is more familiar, more accessible, and easier to provide. Healthcare systems are now at a crossroads. Some will continue to build on their virtual care platforms to benefit their patients and advance their organizational goals. Others will backslide to business as usual, sacrificing their recently hard-earned gains and jeopardizing their long-term positions.

## Why deliver virtual care?

Prior to the pandemic, many healthcare systems achieved high operating margins through care delivered almost exclusively in-person^4,5^. So, why not just return to the pre-pandemic normal? First, the pandemic has accelerated the shift of all sorts of services and activities to home, including remote work, distance learning, online entertainment, eCommerce, and home food delivery^6^. Likewise, countless patients have now received care from home and, given their positive experiences, most would like to continue^7^. Health systems that respond to this consumer demand may grow their local market share and reach into new geographic regions. Those that do not may lose business to their usual competitors, as well as an increasing number of new entrants.

Second, virtual care may reduce costs and improve outcomes^8^. For example, the Hospital at Home program developed at Johns Hopkins uses a combination of in-person home care, remote monitoring, and video visits to enable select patients to receive hospital-level care at home. Compared to traditional hospitalizations, the program reduces costs by one-third, improves patient and family satisfaction, and delivers equivalent patient outcomes^9^.

Third, virtual care may increase care access. After UC San Francisco instituted an eConsult program, the proportion of patients who received specialty care within 2 weeks jumped by 17%^10^. Likewise, UC Davis has long used video visits to extend care to patients who are unable to travel to its campus^11^.

Finally, health systems may use virtual care to better integrate and leverage resources across their hospitals and clinics. At UPMC, two full-time infectious disease specialists use e-consults, video consults, and physician-to-physician phone consults to care for inpatients at 13 system hospitals^12^. Similarly, academic specialists at Mass General Brigham provide peer-to-peer consults to 30 affiliated hospitals across New England, allowing 60% of patients to remain in the community site rather than being transferred to their academic medical centers, which are often at capacity^13^.

## How to deliver virtual care?

Virtual care is not a type of medicine. Rather, it is a set of tools for delivering care and improving health at a distance, both synchronously (e.g., video and phone visits) and asynchronously (e.g., eVisits, messaging, remote monitoring, and eConsults)^14^. Having rushed to implement these tools, health systems are now challenged to make them maximally useful and usable for patients, clinicians, and staff—otherwise, care will simply revert to the in-person status quo. First, virtual care must meet real needs. For patients, this means a more convenient, timely, affordable, and/or effective path to better health. For clinicians, this means better patient care, more flexible and efficient work, additional referrals, and/or higher pay. Second, making virtual care easy to use requires intuitive, user-friendly, and accessible technology, with agile teams readily available to troubleshoot any technical issues. Just as important are streamlined workflows, including ways patients and non-clinical staff can quickly determine when virtual care is available and appropriate, as well as seamless links to services that must still happen in-person, such as medication administration, immunizations, diagnostic tests, and procedures.

In response to the pandemic, most health systems have focused on implementing virtual tools as a substitute for in-person care, most notably video rather than clinic visits. To unlock virtual care’s full potential, they must ultimately go further and make it a central element of care design. A useful analogy is personal banking. Whereas previously almost all services required customers to visit a branch and interact with a bank employee, today the typical consumer performs basic transactions using online or mobile applications, withdraws cash at ATMs, and only steps inside a physical branch for more complex activities^15^.

Health systems should develop similar “multi-channel” strategies that blend traditional in-person care with asynchronous (online and mobile self-service tools, remote monitoring, secure messaging, and eConsults) and synchronous (phone and video visits) virtual options. The goal is to match patients with the channel(s) that best meet their needs and preferences at the time^16^. Care for certain conditions (e.g., behavioral health) may shift to primarily virtual channels, care for others (e.g., orthopedics) may remain predominantly in-person, and care for most may span different channels over time, with virtual complementing in-person care, and vice versa. Importantly, redesigning care in this way requires reconfiguring care teams and modifying individual team member roles and routines^17,18^.

Many health systems are already moving in this direction. At Sutter Healthcare, patients experiencing acute, low acuity conditions may start with a chatbot, which, based on the history and symptoms they report, guides them to self-care, a video visit, or in-person care at a nearby facility^19^. Mayo Clinic’s OB Nest program provides low-risk pregnant women tools to monitor and transmit their health data from home, along with phone or online access to an assigned nurse. The program reduces in-person pre-natal visits from the standard 14 to 8, decreases stress, and increases satisfaction^20^. And at Kaiser Permanente, primary care teams (one physician, medical assistant, and pharmacist) use technology (disease registries, wearable devices, voice and text messages, and video functionality) to extend care to patients with chronic conditions where they actually live their lives, thereby enhancing experiences, reducing costs, and improving health outcomes^21^.

## How to sustain virtual care?

As is commonly said in healthcare, “no margin, no mission.” Health systems are challenged to develop business models that can sustain their virtual services. Most health systems derive the bulk of their revenue through fee-for-service (FFS) reimbursement^22^ which, prior to the pandemic, was quite limited for virtual care. Since then, the federal government, state governments, and private payers have relaxed restrictions and increased virtual care coverage and payment. New federal and state laws will be needed to extend many of these changes beyond the public health emergency^23^. Even then, virtual care reimbursement may remain less than in-person reimbursement, may not always include facility fees, and may be less likely to result in downstream revenue from lab or imaging tests that would otherwise have been performed within the office.

Virtual care may fit better under alternative (non-FFS) risk-based payment models, which create incentives and increase flexibility to redesign care in ways that best meet patient needs. A case in point is Kaiser Permanente, which since 2017 has performed more than half of all visits virtually^24^. Other systems may target virtual care to the populations they serve through their accountable care organization and bundled payment programs, as well as through contracts with Medicare Advantage organizations, Medicaid managed care organizations, and self-insured employers^25^. Last, virtual care presents opportunities to partner with employers, payers, smaller health systems, retail providers, risk-based primary care practices, and digital health providers.

Irrespective of the revenue model, virtual care may enable health systems to reduce costs. Though it may initially require as much or even more staff time than in-person care, efficiency should improve over time, and a shift to more self-service could drive productivity^26^. Likewise, systems that move enough in-person care to virtual may be able to reduce their footprint, or at least reconfigure their space to be more flexible and less costly.

## Guarding against unintended consequences

Technological advances bring benefits along with unanticipated side effects^27^. Virtual care is no exception. First, by virtue of its convenience, virtual care may be overused and paradoxically increase spending, such as if patients use it for trivial problems they would have otherwise managed on their own^28^. Strategies will be needed to limit any such “supplier-induced demand”^29^. Second, virtual care may be underused by key segments of the population, including those with limited English proficiency, low digital literacy, vision and hearing impairment, and poor access to the internet and digital devices. Health systems may somewhat bridge this digital divide by helping their vulnerable patients access necessary technology at home or at nearby community hubs, providing adequate technical support and language interpreters, deploying community health workers, and offering low-tech care options, such as telephone visits. Third, if misused, virtual care may harm patients. Health systems may mitigate this risk by developing protocols for what can and cannot be treated virtually, applying quality improvement methods, and training clinicians so they feel confident providing care virtually and are able to recognize the limits of doing so. Fourth, by making clinicians more accessible to patients and forcing them to spend even more time managing technology and staring at screens, virtual care may contribute to burnout. Preventing this will require support staff and/or software algorithms to sort signals from noise, and training to help clinicians best preserve their human connections with their patients^30^. Overall, health systems should evaluate the virtual care they deliver to ensure it is safe, timely, efficient, effective, equitable, and patient-centered^31^.

## Closing

The pandemic forced healthcare systems to rapidly expand their virtual care offerings. As the pandemic stabilizes and eventually recedes, many will be tempted to completely roll back to traditional in-person care. However, virtual care is no longer optional. It is now a basic expectation and a much-needed opportunity to advance high-value, technology-enabled, person-centered care. Although change is never easy and the benefits may be delayed, health systems must continue to build and implement useful and usable virtual solutions, then integrate them into their broader care and operational models in ways that benefit their organizations and the communities they serve.
